# Determination of radionuclides and radiochemical impurities produced by in-house cyclotron irradiation and subsequent radiosynthesis of PET tracers

**DOI:** 10.1007/s12149-016-1134-3

**Published:** 2016-10-15

**Authors:** Kiichi Ishiwata, Kunpei Hayashi, Masanari Sakai, Sugio Kawauchi, Hideaki Hasegawa, Jun Toyohara

**Affiliations:** 1 Research Team for Neuroimaging, Tokyo Metropolitan Institute of Gerontology, Tokyo, Japan; 2 Institute of Cyclotron and Drug Discovery Research, Southern TOHOKU Research Institute for Neuroscience, 7-115 Yatsuyamada, Koriyama, 963-8052 Japan; 3 Department of Biofunctional Imaging, Fukushima Medical University, Fukushima, Japan; 4 Production Technology Development Department, FUJIFILM RI Pharma, Sammu, Japan

**Keywords:** PET tracers, Quality control, Radionuclides, Radiochemicals

## Abstract

**Objective:**

To elucidate the radionuclides and radiochemical impurities included in radiosynthesis processes of positron emission tomography (PET) tracers.

**Methods:**

Target materials and PET tracers were produced using a cyclotron/synthesis system from Sumitomo Heavy Industry. Positron and γ-ray emitting radionuclides were quantified by measuring radioactivity decay and using the high-purity Ge detector, respectively. Radiochemical species in gaseous and aqueous target materials were analyzed by gas and ion chromatography, respectively.

**Results:**

Target materials had considerable levels of several positron emitters in addition to the positron of interest, and in the case of aqueous target materials extremely low levels of many γ-emitters. Five ^11^C-, ^15^O-, or ^18^F-labeled tracers produced from gaseous materials via chemical reactions had no radionuclidic impurities, whereas ^18^F-FDG, ^18^F-NaF, and ^13^N-NH_3_ produced from aqueous materials had several γ-emitters as well as impure positron emitters. ^15^O-Labeled CO_2_, O_2_, and CO had a radionuclidic impurity ^13^N-N_2_ (0.5–0.7 %).

**Conclusions:**

Target materials had several positron emitters other than the positron of interest, and extremely low level γ-emitters in the case of aqueous materials. PET tracers produced from gaseous materials except for ^15^O-labeled gases had no impure radionuclides, whereas those derived from aqueous materials contained acceptable levels of impure positron emitters and extremely low levels of several γ-emitters.

**Electronic supplementary material:**

The online version of this article (doi:10.1007/s12149-016-1134-3) contains supplementary material, which is available to authorized users.

## Introduction

The quality assurance of positron-emitting tracers used in positron emission tomography (PET) is performed in accordance with guidance documents such as United States Pharmacopeia/National Formulary (USP/NF) and European Pharmacopeia (EP). Although slight differences among the documents were discussed previously [1], basic requirements include characters, radionuclidic identity, radionuclidic purity, radiochemical purity, chemical purity, pH, residual solvents, bacterial endotoxin, and sterility. Most tests can be finished before the release of PET tracers; however, tests such as those for sterility and endotoxins, especially in the case of ^11^C-tracers, and radionuclidic purity depending on the measurement methods are completed after the release.

Regarding to radionuclidic purity, γ-ray spectrometry is required for the detection and quantification of impurities. For the preparation of fludeoxyglucose (^18^F) injection (^18^F-FDG), sodium fluoride (^18^F) injection (^18^F-NaF), and ammonia (^13^N) injection (^13^N-NH_3_), the radionuclides ^18^F and ^13^N are usually produced by proton irradiation of ^18^O-H_2_O and ^16^O-H_2_O, respectively, and it is well known that these aqueous target solutions contain very small amounts of γ-emitters with a longer half-life than ^18^F (109.8 min) [2 and references therein]. Most of these impurities are excluded from the final injections, but some impurities potentially remained in the preparation processes without purification using distillation or high-performance liquid chromatography (HPLC). Their levels may be much lower than that of ^18^F or ^13^N of interest, and the criteria for radionuclidic purity of PET tracers such as ^18^F-FDG and ^18^F-NaF required by the USP/NF (no less than 99.5 %) [3, 4] and EP (minimum 99.9 %) [5, 6]. Radionuclidic purity is also required for ^11^C-labeled compounds such as ^11^C-flumazenil and ^11^C-raclopride; however, it is hardly considered that these tracers derived from ^11^C-CO_2_ via the chemical reactions and HPLC purification may contain radionuclides other than ^11^C. The requirement for radionuclidic identity and purity for PET tracers synthesized chemically from gaseous materials seems to be substantially meaningless from a scientific point of view. However, systematic studies on what kinds of radionuclides and radiochemical species are included in the radiosynthesis processes of many PET tracers have not been reported.

The aim of this study was to clarify the radionuclides included in the radiosynthesis processes from target materials into final products. We focused on PET tracers labeled with four conventional radionuclides, ^11^C, ^13^N, ^15^O, and ^18^F, and investigated radionuclides and radiochemical species in the target materials, positron-labeled precursors, and five PET tracers. The target materials included ^11^C-, ^15^O-, and ^18^F-labeled gases and ^13^N- and ^18^F-target solutions. The radionuclides investigated were short half-life positron emitters and longer half-life γ-emitters. We discuss the significance of examinations of radionuclidic identity and purity in the quality control of PET tracers.

## Materials and methods

### General

We used a 20 MeV cyclotron (CYPRIS HM-20), target and synthesis system developed by Sumitomo Heavy Industry (Tokyo, Japan). Target folders and materials used and the expected nuclear reactions are summarized in Supplementary Tables 1 and 2, respectively. The production of positron emitters and their labeled compounds were according to the standard specifications of Sumitomo Heavy Industry. Because the radionuclidic identity and purity could be changed depending on several parameters such as irradiation time, beam current, and target materials, we set the integrated beam currents to be suitable for routine production of each PET tracers in clinical use, and changed depending on the analyses to accurately evaluate or to avoid unnecessary radiation dose. The detailed information is summarized in Supplementary tables.

### Production of ^11^C-CO_2_, ^11^C-methyl iodide, and ^11^C-methylated compounds

^11^C-CO_2_ was produced by proton irradiation of N_2_ containing 0.5 % O_2_ at a pressure of 0.8 MPa.

#### ^*11*^*C*-*CO*_*2*_*gas preparation*

After 15-min irradiation with 10–50 μA, ^11^C-CO_2_ target gas was passed through a coiled stainless steel tube [0.5 mm inner diameter (i.d.) × 90 cm length] immersed in liquid Ar (boiling point −186 °C) to trap ^11^C-CO_2_ (boiling point −196 °C). After returning the stainless steel tube to room atmosphere, the ^11^C-CO_2_ gas was recovered in a Tedlar^®^ bag for about 60 s with a 30 ml/min N_2_ flow to measure radioactivity decay.

#### *Syntheses of*^*11*^*C*-*methyl iodide and three*^*11*^*C*-*methylated tracers*

After 15-min irradiation with 5–50 μA, ^11^C-CH_3_I and ^11^C-methylated compounds were prepared using a multipurpose synthesizer CFN-MPS100 (Sumitomo Heavy Industries). ^11^C-CO_2_ gas was purged with a 30 ml/min N_2_ flow into 0.1 ml of 0.1 M LiAlH_4_ in tetrahydrofuran (ABX, Radeberg, Germany). After removal of tetrahydrofuran using a 200 ml/min N_2_ flow heated to 180 °C, 0.5 ml of HI was added and the solution was heated for 45 s. The ^11^C-CH_3_I produced was recovered in a Tedlar^®^ bag for about 60 s with a 30 ml/min N_2_ flow to measure radioactivity decay. Radiosyntheses of ^11^C-methionine, ^11^C-ITMM, and ^11^C-CB184 are described in the Supplementary material.

### Production of ^15^O-labeled gases and H_2_O

^15^O-Gas was produced by deuteron irradiation of N_2_ containing 0.5 % CO_2_ or 2 % O_2_ at a pressure of 0.29 MPa. After 10-min irradiation with 10–30 μA, ^15^O-gas under continuous irradiation was transferred to a ^15^O-gas/water synthesis system CYPRIS-G (Sumitomo Heavy Industries) with a 300 ml/min flow to produce the three ^15^O-gases described below.

#### ^*15*^*O*-*CO*_*2*_*production*

Target ^15^O-gas containing 0.5 % CO_2_ was passed through a crushed carbon granules (size, between 1.0 and 3.35 mm) column (9.8 mm i.d. × 150 mm length) at 400 °C to convert traces of ^15^O-CO to ^15^O-CO_2_.

#### ^*15*^*O*-*O*_*2*_*production*

Target ^15^O-gas containing 2 % O_2_ was passed through three columns of crushed carbon granules (size, between 1.0 and 3.35 mm, 9.8 mm i.d. × 150 mm length), soda lime (9.0 mm i.d. × 150 mm length) and molecular sieve 5A 1/16 (Wako Pure Chemical Industries, Tokyo, Japan; 9.8 mm i.d. × 150 mm length) at room temperature. Traces of ^15^O-CO and ^13^N-N_2_O were removed by these columns.

#### ^*15*^*O*-*CO production*

Target ^15^O-gas containing 2 % O_2_ was passed through two columns of charcoal (9.8 mm i.d. × 150 mm length) heated at 1000 °C and soda lime (9 mm i.d. × 150 mm length) at room temperature. Traces of ^15^O-CO_2_ are removed using the soda lime.

#### ^*15*^*O*-*H*_*2*_*O synthesis*

^15^O-O_2_ gas produced above was passed through a palladium black (about 20 mg, Wako Pure Chemical Industries) column (9.0 mm i.d. × 2 mm length) heated at 130 °C and a molecular sieve 5A 1/16 column (9.0 mm i.d. × 70 mm length) at room temperature. This gas at a flow rate of 500 ml/min is mixed with H_2_ at a flow rate of 7 ml/min and passed over a Pd wire (Aldrich 267163, Atlanta, GA) column (9.0 mm i.d. × 15 mm length) heated at 180 °C, and then bubbled into a sterile vial containing physiological saline.

### Production of ^18^F-F_2_ and 4-^10^B-borono-2-^18^F-fluoro-l-phenylalanine (^18^F-FBPA)

^18^F-F_2_ was produced by deuteron irradiation (30 min for analysis and 120 min for production of ^18^F-FBPA) of neon gas containing 0.6 % F_2_ gas at a pressure of 0.3 MPa (Supplementary Table 1). Radiosynthesis of ^18^F-FBPA is described in the Supplementary data.

### Production of ^13^N-ammmonium and ^13^N-NH_3_

^13^N-Ammonium solution was produced by proton irradiation of natural water containing 10 mM ethanol at a pressure of 1.8 MPa [7].

#### ^*13*^*N*-*NH*_*3*_*production*

^13^N-NH_3_ was produced using a ^13^N-NH_3_ synthesizer N100 (Sumitomo Heavy Industries). After 10-min irradiation with 10–50 μA, ^13^N-ammonium solution was passed through a Sep-Pak Accel Plus CM Plus Short cartridge (Waters, Milford, MA). The cartridge was washed with 10 ml water for injection twice, and the ^13^N-ammonium was eluted with 12 ml physiological saline for injection.

### Production of ^18^F-fluoride, ^18^F-NaF, and ^18^F-FDG

^18^F-Fluoride solution was produced by proton irradiation of enriched ^18^O-water (^18^O: ≥98.0 atom %, Taiyo Nippon Sanso, Tokyo, Japan) at a pressure of 1.8 MPa.

#### ^*18*^*F*-*NaF production*

^18^F-NaF was prepared using a CFN multipurpose synthesizer CFN-MPS100 (Sumitomo Heavy Industries). After 3–45 min irradiation with 5–20 μA, ^18^F-fluoride solution was passed through a Sep-Pak Accel Plus QMA Plus Light cartridge (Waters). The cartridge was washed with 10 ml water for injection, and ^18^F-fluoride was eluted with 1 ml physiological saline for injection and diluted with 14 ml physiological saline.

#### ^*18*^*F*-*FDG production*

After 7–45-min irradiation with 20–50 μA, ^18^F-FDG was prepared using an ^18^F-FDG synthesizer F300 (Sumitomo Heavy Industries).

### Determination of positron emitters in target materials and radiolabeled compounds

Immediately after the end of bombardment (EOB), gaseous and aqueous target materials were recovered in a Tedlar^®^ bag for gases and in a small glass vial for liquids, respectively, and the radioactivity decay was successively measured at first short intervals (15, 20, 30, and 60 s), then intermediate intervals (2, 5, 10, and 20 min) and finally long intervals (0.5, 1, and 2 h) using a radioisotope calibrator CRC^®^-712 (Capintec, Ramsey, NJ). Immediately after the recovery of ^11^C-CO_2_ and ^11^C-methyl iodide gases and synthesis of radiolabeled compounds, the radioactivity decay was also successively measured, and percentages of radionuclides were decay-corrected at the EOB, at the time of recovery, or the end of synthesis (EOS).

### Gas chromatography

Radiolabeled gases were analyzed using a gas chromatography (GC) system mounted in a ^15^O-gas/water synthesis system CYPRIS-G (Sumitomo Heavy Industries). Radiolabeled gases were analyzed on a Porapak Q column (50/80 mesh, 3 mm i.d. × 4 m length, Shimadzu, Kyoto, Japan) and a molecular sieves 13X column (30/60 mesh, 3 mm i.d. × 4 m length, Shimadzu) at 55 °C with a He flow at 55 kPa pressure (about 110 ml/min). CO_2_ and N_2_O were absorbed using the molecular sieves 13X.

### Ion chromatography

^13^N-ammonium and ^18^F-fluoride target solutions, ^13^N-NH_3_, ^18^F-NaF, and ^18^F-FDG were successively analyzed 3–5 times using an ion chromatography (IC) system (Prominence HIC-SP, Shimadzu) immediately after EOS (2–8 min) up to 73 min to assign radionuclides of radioactive peaks. For analysis of anion a Shim-pack IC-SA2 (4.6 mm i.d. × 250 mm length, Shimadzu) was used at 30 °C by elution with of 12 mM NaHCO_3_/0.6 mM Na_2_CO_3_ = 1/1 at a flow rate of 1.0 ml/min using an electric conductivity detector (suppressor method) and radioactivity monitor. For analysis of cations a Shim-pack IC-C4 (4.6 mm i.d. × 150 mm length, Shimadzu) was used at 40 °C by elution with of 3.5 mM oxalic acid/1 mM 18-Crown-6 ether = 1/1 at a flow rate of 1.0 ml/min by a non-suppressor method.

### Detection of long half-lived gamma emitters in positron emitting compounds

For analyses of γ-emitters of PET tracers we set the integrated beam currents to be suitable for routine clinical use: ^13^N-NH_3_, 10-min irradiation with 20 μA; ^18^F-FDG, 45-min irradiation with 50 μA; ^11^C-methionine, 15-min irradiation with 10 μA; ^18^F-FBPA, 120-min irradiation with 25 μA. The irradiation condition of ^18^F-fluoride target solution and ^18^F-NaF was same as that of ^18^F-FDG. ^13^N-ammonium target solution was produced by 30-min irradiation with 50 μA. Target materials and radiolabeled compounds were diluted with H_2_O to a 100 ml total volume, and the presence of impure metal ions emitting γ-rays was measured using a high-purity Ge detector at FUJIFILM RI Pharma (GMX20190-P, SEIKO EG&G, Tokyo, Japan) for ^18^F-target materials and ^18^F-labeled compounds and at Tokyo Nuclear Services (GEM20200, SEIKO EG&G) for others. The radionuclides evaluated included 15 γ-emitters: ^22^Na, ^48^V, ^51^Cr, ^52^Mn, ^54^Mn, ^55^Co, ^56^Co, ^57^Co, ^58^Co, ^57^Ni, ^67^Ga, ^93m^Mo, ^95^Tc, ^96^Tc, and ^181^Re. Each sample was measured twice for 1 and 10 h at 2–3 and 3–11 days, respectively, after EOB.

## Results and discussion

### Positron-emitting radionuclides and radiochemicals in ^11^C-CO_2_ target gas and ^11^C-labeled compounds

**Fig. 1:**
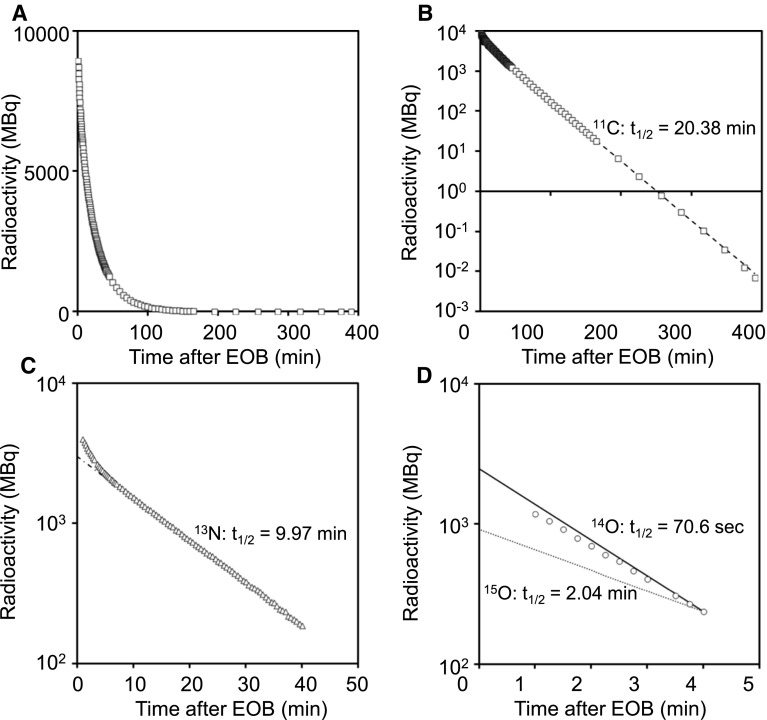
Radioactivity decay curves of ^11^C-CO_2_ target gas. **a** Total radioactivity decay (*square*) on a linear scale. **b** Total radioactivity decay (*square*) on a log scale indicates a half-life of ^11^C. The ^11^C-radioactivity was extrapolated to time zero and subtracted from total radioactivity. **c** The residual radioactivity decay (*triangle*) on a log scale indicates a half-life of ^13^N. The ^13^N-radioactivity was further subtracted from the residual radioactivity. **d** The final radioactivity decay (*circle*) on a log scale indicates a half-life of ^14^O

The total radioactivity decay curve of ^11^C-CO_2_ target gas (*n* = 3) is plotted in Fig. 1. There were three phases on a log scale. The decay line between 30 and 400 min (Fig. 1b) was well fitted to the decay of ^11^C (*t*_1/2_ = 20.4 min). After subtracting ^11^C-radioactivity extrapolated to time zero at EOB from total radioactivity, the residual radioactivity decay line between 10 and 40 min fitted well to the decay of ^13^N (*t*_1/2_ = 9.97 min) (Fig. 1c). When further subtracting ^13^N-radioactivity as above, the decay line between 1 and 4 min was slightly lower than the decay line of ^14^O (*t*_1/2_ = 70.6 s) (Fig. 1d). However, when considering the ratios of isotope: ^14^N vs ^15^N vs ^16^O in the target material and the cross section of each isotope: ^14^N(p, n)^14^O vs ^15^N(p, n)^15^O vs ^16^O(p, d)^15^O, we concluded that Fig. 1d showed the decay of ^14^O (*t*_1/2_ = 70.6 s) rather than that of ^15^O (*t*_1/2_ = 2.04 min). Then, from the radioactivities of ^11^C, ^13^N, and ^14^O at EOB the ratios of each radioisotope were calculated as ^11^C, 47.3 %: ^13^N, 28.5 %: and ^14^O, 24.2 % (Supplementary Table 3). Radioactivity decay curves plotted on a log scale for ^11^C-CO_2_ gas, ^11^C-CH_3_I, ^11^C-methionine, ^11^C-ITMM, and ^11^C-CB184, showed only one component ^11^C (Supplementary Table 3).

**Fig. 2:**
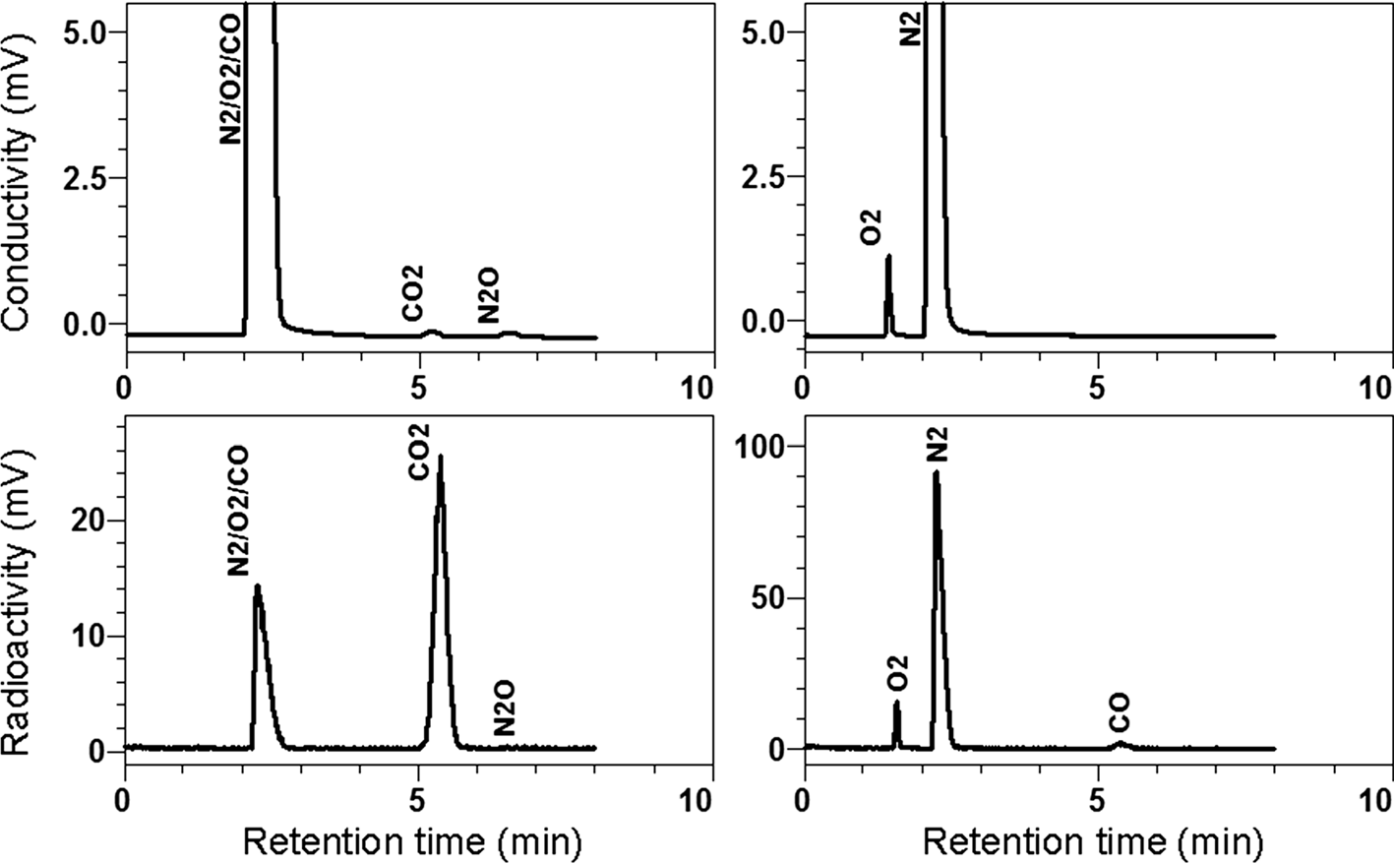
Gas chromatograms of ^11^C-CO_2_ target gas. ^11^C-CO_2_ target gas was directly applied to a gas chromatography system mounted in a ^15^O-gas/water synthesis system CYPRIS-G (Sumitomo Heavy Industries). *Left side* analysis using a Porapak Q column; *right side* analysis using a Molecular Sieve 13X column. *Upper row* thermal conductivity; *lower row* radioactivity

^11^C-CO_2_ target gas was analyzed by GC (*n* = 4). Radioactive peaks were detected in the retention times of O_2_/N_2_/CO, (2.4 min), CO_2_, (5.4 min) and N_2_O (6.5 min) on Porapak Q and those of O_2_, (1.5 min), N_2_, (2.2 min), and CO (5.3 min) on Molecular Sieve 13X (Fig. 2). When considering radionuclides detected by measurement of radioactivity decay, we assigned that CO_2_ (average 60.4 %) and CO (1.0 %) were labeled with ^11^C, N_2_ (33.6 %) and N_2_O (0.1 %) were with ^13^N, and O_2_ (4.4 %) was ^14^O (Supplementary Table 4).

We did not analyze ^11^C-CO_2_ gas condensed in a stainless steel tube in liquid Ar by GC. However, the radioactivity decay of the ^11^C-CO_2_ gas showed a single component. ^13^N-N_2_ and ^11^C-CO in the ^11^C-CO_2_ target gas were not trapped and ^14^O-O_2_ was hardly trapped at liquid Ar temperature when considering the difference of boiling points: N_2_, −196 °C; CO, −192 °C; Ar, −186 °C; O_2_, −183 °C. A trace of ^13^N-N_2_O (0.1 %, boiling point of N_2_O, −88 °C) might lower than the detection limit during the condensation process of the ^11^C-CO_2_ target gas. Thus, we concluded that radionuclidic pure ^11^C-CO_2_ was used for the ^11^C-labeling of PET tracers after the condensation process of the ^11^C-CO_2_ target gas at liquid Ar temperature.

### Positron-emitting radionuclides and radiochemicals in ^15^O-gases and ^15^O-H_2_O

Radioactivity decay of ^15^O-CO_2_, ^15^O-O_2_, and ^15^O-CO (*n* = 4 for each) and ^15^O-H_2_O (*n* = 3) was measured for about 130 min until reaching to background levels (Supplementary Fig. 1 for ^15^O-CO_2_). All three ^15^C-gases showed a biphasic decay curve with *t*_1/2_ = 10 and 2 min. Contamination by ^13^N in 12 preparations of three ^15^C-gases was 0.5–0.7 % of total radioactivity at EOB (Supplementary Table 5). ^15^O-H_2_O decayed away with *t*_1/2_ = 2 min.

^15^O-Gases were analyzed using GC (Supplementary Fig. 2). ^15^O-CO_2_ (*n* = 6), ^15^O-O_2_ (*n* = 3), and ^15^O-CO (*n* = 6) included 0.5 % ^15^O-CO plus 0.7 % ^13^N-N_2_, 0.6 % ^13^N-N_2_, and 0.7 % ^13^N-N_2_, respectively, as radioactive impurities (Supplementary Table 6). Similar radioactive impurities in each of the ^15^O-gases were found by several groups [8 and references therein]; however, our method could exclude radiolabeled N_2_O using Molecular Sieve 13X.

We did not analyze ^15^O-H_2_O by GC. However, radioactive contamination in the ^15^O-H_2_O cannot be considered, because inert ^13^N-N_2_ was completely removed in the synthesis process of ^15^O-H_2_O using ^15^O-O_2_.

Detection of a radionuclidic impurity ^13^N_2_ in a range of 0.5–0.8 % of total radioactivity in all three ^15^O-gases indicates that criteria are necessary for both radionuclidic and radiochemical impurities in quality assurance of ^15^O-gases.

### Positron-emitting radionuclides and radiochemicals in ^18^F-F_2_ target gas and ^18^F-FBPA

Radioactivity decay of ^18^F-F_2_ target gas (*n* = 3) showed a biphasic decay curve (Supplementary Fig. 3). The decay line up to 1300 min fitted well to the decay of ^18^F, and that between 1.5 and 4 min fitted well to the decay of ^23^Ne (37.2 s). The amount of ^23^Ne (83.2 %) was much larger than that of ^18^F-F_2_ (16.8 %) at EOB (Supplementary Table 7). ^23^Ne is inert for chemical reactions. Radioactivity decay of ^18^F-FBPA showed only a component of ^18^F. In the present study, we added F_2_ at 0.6 % in Ne which was sufficient to prevent adsorption of ^18^F-F_2_ on the inner surface of the target and tubing for transfer to the synthesis system. We consider that examination of radionuclidic impurity could be omitted in the quality assurance of ^18^F-labeled PET tracers derived from ^18^F-F_2_.

### Positron-emitting radionuclides and radiochemicals in ^13^N-ammonium target solution and ^13^N-NH_3_

Radioactivity decay of an ^13^N-ammonium target solution (*n* = 3) showed a three-phase curve (Supplementary Fig. 4). The decay line between 100 and 1200 min fitted well to the decay of ^18^F, and those between 10 and 70 min and between 3 and 8 min fitted well to the decay of ^13^N and ^15^O, respectively, and the ratios of ^13^N, ^15^O, and ^18^F were 58.3, 41.1, and 0.6 %, respectively, at EOB (Supplementary Fig. 4; Table 8). It should be noted that during the recovery of ^13^N-ammonium target solution in a glass vial by He pressure, radioactive gases such as ^13^N-N_2_ [7] could be removed, if they were present in the target solution. We detected ^15^O and ^18^F as contaminants in the ^13^N-ammonium target solution as reported previously [9–11]. On the other hand, Tornai et al. found ^17^F (*t*_1/2_ = 64.5 s) in addition to ^15^O in an ^13^N-ammonium target solution produced by proton irradiation with an incident energy of 21 MeV, but not with an incident energy of 10.7 MeV [12].

^13^N-NH_3_ (*n* = 3) showed a biphasic decay curve with *t*_1/2_ = 10 and 110 min, and the ratio of ^18^F was 0.005 % of total radioactivity at EOS (Supplementary Fig. 5; Table 8). It was pointed out that the elapsed time when the ratio of ^18^F in each of three ^13^N-NH_3_ preparations exceeded 0.1 % of ^13^N was 41–51 min after EOS.

**Fig. 3:**
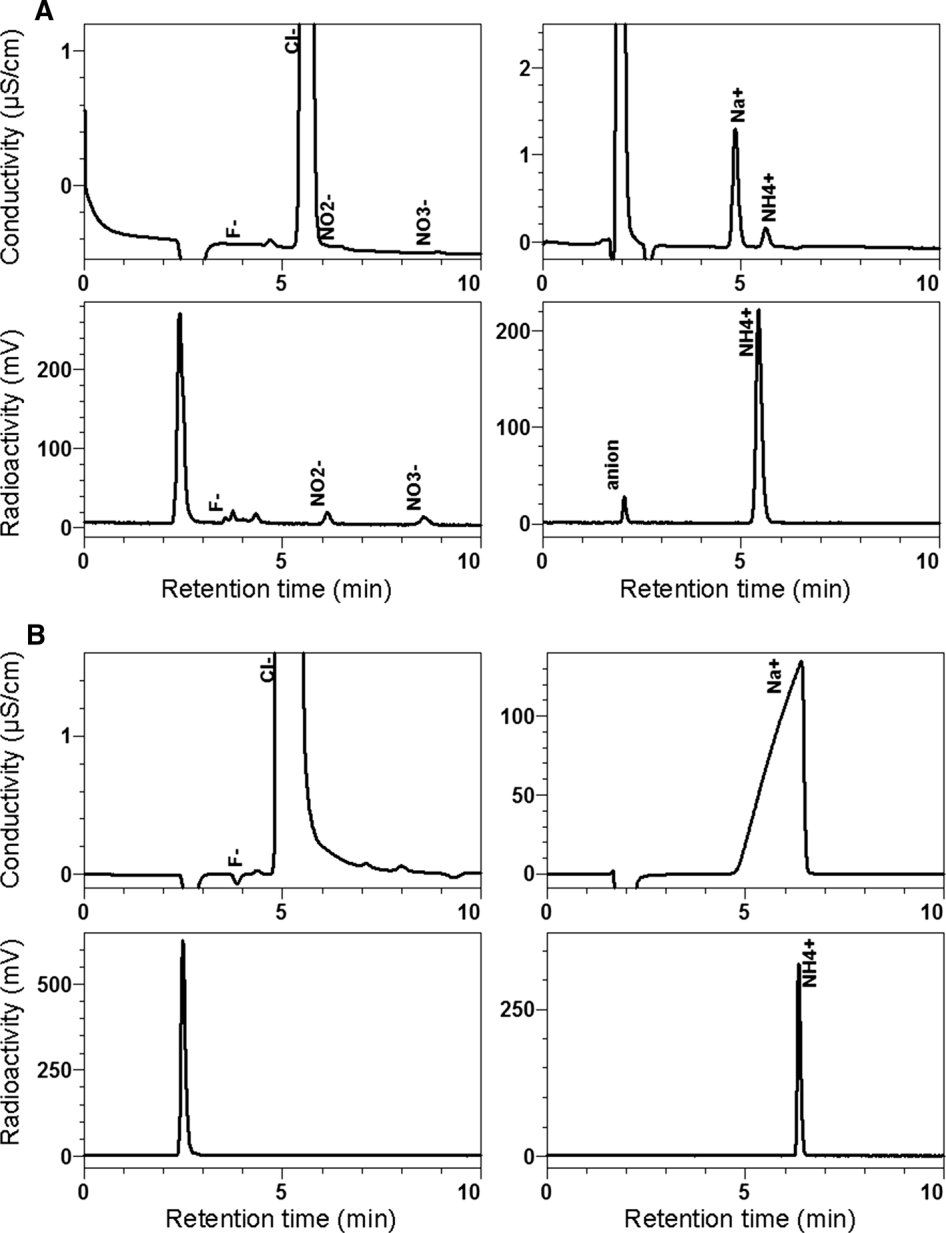
Ion chromatograms of ^13^N-ammonium target solution (**a**) and ^13^N-NH_3_ (**b**). *Left side* analysis on an anion-exchange Shim-pack IC-SA2 column; *right side* analysis using a cation-exchange Shim-pack IC-C4 column. *Upper row* conductivity; *lower row* radioactivity

^13^N-Ammonium target solution and ^13^N-NH_3_ were analyzed using IC (*n* = 3). In the first of successive analyses of the ^13^N-ammonium target solution on an anion-exchange column, more than four minor radioactive peaks, in addition to a main radioactive peak in the void volume, were detected (Fig. 3a). The retention times of the three peaks were consistent with those of fluoride (3.9 min), nitrite (4.3 min), and nitrate (8.9 min). Based on previous studies [7, 11, 13, 14], the nitrite and nitrate were probably labeled with ^13^N. Candidate compounds for other minor radioactive compounds (void-1 and unknown-2) may be ^13^N-NH_2_OH [14] and ^15^O-H_2_O [12]. We detected ^15^O in the ^13^N-ammonium target solution by measuring radioactivity decay (Supplementary Table 8); however, we could not assign ^15^O-labeled components in successive analyses. IC may barely detect low levels of ^15^O-labeled components. The percentage of the main peak in the void volume containing ^13^N-ammonium was 96.5 % at EOS, when the contribution of ^15^O as a radioactive contaminant was ignored (Supplementary Table 9). ^13^N-NH_3_ showed a radioactive peak on an anion-exchange column.

In successive analyses of the ^13^N-ammonium target solution on a cation-exchange column, three radioactive peaks were detected for these periods (Fig. 3a). The peak area corresponding to the retention time of ammonium (5.6 min) decreased in accordance with a half-life of ^13^N, but the areas of other two peaks (retention time, 1.8 and 2.1 min) decreased more slowly, indicating that each of two included ^13^N- and ^18^F-chemicals. The 2.0 min component contained of ^18^F-fluoride, as described later. The percentage of the ^13^N-ammonium peak was 92.1 % at EOS on the premise there were no ^15^O-labeled components (Supplementary Table 9).

In ^13^N-NH_3_, only one radioactive peak was detected in the void volume and at the retention time of ammonium on anion- and cation-exchange columns, respectively (Fig. 3b). The contaminant ^18^F, probably ^18^F-fluoride, detected by measuring radioactivity decay (0.005 % of total radioactivity), could not be monitored using a radioactivity detector even in the last of successive analyses (60–65 min at EOB) probably because of the very low level available to detect.

In quality assurance of ^13^N-NH_3_, ^18^F-fluoride is noticed as a radionuclidic impurity but not radiochemical impurity. A limited time of ^13^N-NH_3_ for clinical use may be at longest a three half-life period (30 min) after the EOS. Up to that time the ratio of ^18^F increases, but is a very low level (about 0.05 % of total radioactivity) that is acceptable for clinical use.

### Positron-emitting radionuclides and radiochemicals in ^18^F-fluoride target solution, ^18^F-NaF and ^18^F-FDG

A radioactivity decay curve of ^18^F-fluoride target solution (*n* = 3) showed three decay lines of ^18^F, ^13^N, and ^17^F (*t*_1/2_ = 64.5 s), and the ratios of ^18^F, ^17^F, and ^13^N were 87.2, 12.0, and 0.8 %, respectively, at EOB (Supplementary Fig. 6; Table 10). Radioactivity decay of ^18^F-NaF (*n* = 4) showed two components ^18^F (99.5 %) and ^13^N (0.5 %) (Supplementary Fig. 7), and ^18^F-FDG (*n* = 3) had a component of ^18^F (Supplementary Table 10). Contamination with ^17^F probably disappeared during the preparation periods of ^18^F-NaF (8–11 min) and ^18^F-FDG (about 30 min). The elapsed times when the ratio of ^13^N in each of the four ^18^F-NaF preparations became less than 0.1 % of ^18^F were calculated to be 21–32 min after EOS. In another ^18^F-FDG sample we measured radioactivity decay until complete decay for over 3 days, and confirmed that no radioactivity with a longer half-life than ^18^F was detected using measurement with a radioisotope calibrator.

^18^F-Fluoride target solutions, ^18^F-NaF and ^18^F-FDG were analyzed by IC (Supplementary Fig. 8). Short half-life ^17^F (*t*_1/2_ = 64.5 s) was hardly detected by IC because of the period to the start analysis and the retention time of ^18^F-fluoride. On an anion-exchange column ^18^F-fluoride target solution and ^18^F-NaF showed a minor radioactive peak of nitrate (8.9 min, 0.6 %) and two minor radioactive peaks corresponding to nitrite (4.3 min, 0.2 %) and nitrate (1.5 %), respectively, in addition to ^18^F-fluoride. ^18^F-FDG showed a main peak (2.5 min) and minor unknown and fluoride peaks. Because the ^18^F-fluoride increased slightly in successive analyses (data not shown), it was probably derived from radiolysis of ^18^F-FDG [15].

On a cation-exchange column, a main radioactive peak for all ^18^F-fluoride target solution, ^18^F-NaF, and ^18^F-FDG, was eluted at 2.0 min, and a minor radioactive peak was detected in the ^18^F-fluoride target solution (1.8 min) and ^18^F-FDG (2.3 min). In successive analyses, the 1.8-min peak area decreased faster than a half-life of ^18^F, indicating that the peak included ^13^N- and ^18^F-chemicals. The percentages of each component on both ion-exchange columns are summarized in Supplementary Table 11.

In clinical use of ^18^F-NaF, a preparation time is very short and ^13^N was detected in it as radionulidic impurities. It is preferable that quality assurance or release of ^18^F-NaF for clinical use is delayed for appropriate time after the EOS until ^13^N-radioactivity decays to a negligible level.

### Detection of long half-life γ-emitters in positron-emitting compounds

The presence of impure metal ions emitting γ-rays in aqueous target materials is well known [2 and references therein]. We measured long half-life γ-emitters in aqueous target solution and related compounds using a high-purity Ge detector (Supplementary Table 12).

In the ^13^N-ammonium target solution (average yield = 13.5 GBq, *n* = 3), 11 of 15 γ-emitters investigated were detected in the order of 10^−6^–10^−9^ compared with ^13^N of interest: ^51^Cr, ^52^Mn, ^54^Mn, ^55^Co, ^56^Co, ^57^Co, ^58^Co, ^57^Ni, ^95^Tc, ^96^Tc, and ^181^Re. Most of these nuclides were removed in the synthesis process of ^13^N-NH_3_ (average yield = 7.4 GBq, *n* = 3), and very small amounts of five γ-emitters (^51^Cr, ^52^Mn, ^55^Co, ^56^Co, and ^58^Co) in the order of 10^−13^–10^−14^ compared with ^13^N of interest remaining in ^13^N-NH_3_. Theoretically, ^13^N-ammonium target solution was expected to contain the same γ-emitters as ^18^F-fluoride target solution described below, because we used the same target system: a Nb-body target with a Havar foil. A finding that two γ-emitters, ^67^Ga and ^93m^Mo, were not detected in ^13^N-ammonium target solution was possibly because of the shorter irradiation times. In the production of ^13^N-NH_3_, extremely small amounts of γ-emitters were left, because we limited the irradiation time to a practical level for clinical use of ^13^N-NH_3_.

In ^18^F-fluoride target solution (average yield = 93.8 GBq, *n* = 3), 13 of 15 γ-emitters were detected in the order of 10^−8^–10^−10^ compared with ^18^F of interest: ^51^Cr, ^52^Mn, ^54^Mn, ^55^Co, ^56^Co, ^57^Co, ^58^Co, ^57^Ni, ^67^Ga, ^93m^Mo, ^95^Tc, ^96^Tc, and ^181^Re. Avila-Rodriguez et al. found 11 γ-emitters other than ^54^Mn and ^67^Ga in ^18^F-fluoride target solution using a Nb-body target with a Havar foil [2]. Undetected two γ-emitters ^22^Na and ^48^V were included as potential radionuclides which were present in the production process of PET tracers depending on the target body and foils [the report on the waste of short half-life radionuclides by Japan Radioisotope Association (May 2003, in Japanese)]. For example, ^48^V was detected using a silver-body target with titanium foil [16]. In the synthesis processes of ^18^F-NaF (average yield = 78.3 GBq, *n* = 3), six γ-emitters were under detection, two (^54^Mn and ^56^Co) were reduced, and the other five (^51^Cr, ^93m^Mo, ^95^Tc, ^96^Tc, and ^181^Re) were not reduced. In ^18^F-FDG (average yield = 63.4 GBq, *n* = 3), nine γ-emitters were below the level of detection, two (^56^Co and ^181^Re) remained, and two (^95^Tc and ^96^Tc) were reduced.

We examined contaminant by γ-emitters in PET tracers derived from gaseous target materials: ^11^C-CO_2_ gas via ^11^C-CH_3_I and ^11^C-CH_3_OTf, and ^18^F-F_2_. Selected PET tracers were ^11^C-methionine and ^18^F-FBPA. The former was prepared without HPLC separation. Both tracers showed no γ-emitters. These findings were reasonably well expected, because it was hardly considered that radiolabeled metal contaminants were derived from gaseous target materials.

### Radionuclidic identity and purity in the quality control of PET tracers

In the present study, we investigated radionuclides and radiochemical species in radiosynthesis processes from target materials to PET tracers. In gaseous and aqueous target materials, a couple of positron emitters were found. However, regarding to radionuclidic purity the tracers produced by chemical reactions: ^11^C-labeled methionine, ITMM and CB184, ^15^O-H_2_O, ^18^F-FBPA, and ^18^F-FDG had only one positron emitter of interest. On the other hand, ^15^O-gases, ^18^F-NaF, and ^13^N-NH_3_ which were produced by passing through catalytic columns or ion-exchange cartridges without chemical reactions, contained minor positron impurities in addition to the positron emitter of interest. Regarding to positron impurities, it was noted that measurement of radioactivity decay was more sensitive than IC. IC could not detect ^18^F in ^13^N-NH_3_ and ^15^O-components in ^13^N-target solution. The radionuclidic purity of each of the three ^15^O-gases was less than 99.5 %; however, an impurity, that was inert ^13^N-N_2_, was reasonably acceptable for clinical use, because it interfered in neither measurement of aimed functions/imaging quality nor the welfare of subjects. The radionuclidic impurities of ^18^F-NaF: ^13^N-nitrite and ^13^N-nitrate decreased rapidly to be less than 0.1 % of ^18^F by 21–32 min after EOS as discussed above. Thus, 30–60 min after EOS, the radionuclidic purity of ^18^F-NaF satisfied the acceptance criteria for ^18^F-NaF required by USP/NF (greater than 99.5 %) and EP (minimum 99.9 %). The amounts of γ-emitters found in ^18^F-FDG, ^18^F-NaF, and ^13^N-NH_3_ were extremely low compared with acceptance criteria for the radionuclidic purity required by USP/NF and EP. The ratios of radionuclidic impurities observed in PET tracers could be slightly changed depending on the integrated beam currents; however, we considered that examinations of the radionuclidic identity and purity were not necessarily for quality assurance of PET tracers in routine production when the radionuclides were produced using a specific cyclotron with fixed energy of proton or deuteron beam (CYPRIS HM-20 in this study) and fixed target and synthesis systems, and when once the radionuclides in target materials and final PET tracers were reasonably analyzed from a scientific point of view. Regarding ^15^O-gases, radiochemical impurities ^13^N_2_ in ^15^O-O_2_ and ^15^O-CO corresponded with radionuclidic impurity, and the ratios of radionuclidic impurity in three ^15^O-gas species were 0.5–0.8 % of total radioactivity. The criteria of radionuclidic purity for PET tracers such as these ^15^O-gases should be determined based on quality for the measurement of the aimed functions and welfare of subjects, but not in the same way as other tracers such as ^18^F-FDG and ^18^F-NaF.

## Conclusion

Gaseous target materials used for the production of PET tracers had considerable levels of several short half-life positron emitters in addition to the positron of interest, and aqueous target materials had extremely low levels of many longer half-life γ-emitters in addition to positron emitters. Five tracers produced from gaseous target materials via chemical reactions had no radionuclidic impurities, whereas ^18^F-FDG, ^18^F-NaF, and ^13^N-NH_3_ produced from aqueous target materials had extremely low levels of several γ-emitters as well as impure positron emitters. Three ^15^O-labeled gases had impure positron-emitting ^13^N-N_2_ (slightly over 0.5 %).

## Electronic supplementary material

Below is the link to the electronic supplementary material.

Supplementary material 1 (DOCX 571 kb)
