# Supplementary material for: Determination of radionuclides and radiochemical impurities produced by in-house cyclotron irradiation and subsequent radiosynthesis of PET tracers
Source: Ann Nucl Med. 2016 Oct 15;31(1):84–92. doi: 10.1007/s12149-016-1134-3 (PMC5233741; doi:10.1007/s12149-016-1134-3)
Supplement: Supplementary file 1 — Supplementary material 1 (DOCX 571 kb) [file 12149_2016_1134_MOESM1_ESM.docx]

**Supplementary material**

**Determination of radionuclides and radiochemical impurities produced by in-house cyclotron irradiation and subsequent radiosynthesis of PET tracers**

Kiichi Ishiwata^a,b,c^*, Kunpei Hayashi^a^, Masanari Sakai^a^, Sugio Kawauchi^d^, Hideaki Hasegawa^d^, Jun Toyohara^a^

^a^Research Team for Neuroimaging, Tokyo Metropolitan Institute of Gerontology, Tokyo, Japan
^b^Institute of Cyclotron and Drug Discovery Research, Southern TOHOKU Research Institute for Neuroscience, Koriyama, Japan

^c^Department of Biofunctional Imaging, Fukushima Medical University, Fukushima, Japan

^d^Production Technology Development Department, FUJIFILM RI Pharma, Sammu, Japan

Address correspondence to: Kiichi Ishiwata, Ph.D., Institute of Cyclotron and Drug Discovery Research, Southern TOHOKU Research Institute for Neuroscience, 7-115 Yatsuyamada, Koriyama, 963-8052, Japan; Phone: +81-24-934-5322; Fax: +81-24-922-5320; E-mail: kiichiishiwata@gmail.com

**Radiosyntheses of positron emission tomography (PET) tracers**

*^11^C-Methionine:* After 15-min irradiation, ^11^C-CH_3_I prepared above was converted to ^11^C-methyl triflate by passing through a silver triflate column (4 mm i.d. × 25 mm length) heated at 200ºC with a 30 ml/min N_2_ flow, and the ^11^C-methyl triflate was trapped in a mixture of 2 ml acetone and 0.5 ml 0.2 M NaOH containing 1–1.5 mg L-homocysteine thiolactone. After adding 1.1 ml 0.1 M HCl, the reaction mixture was evaporated to dryness and the residue was dissolved in 10 ml physiological saline containing 0.5 ml 250 mg/ml ascorbate injection (Nipro Pharma, Osaka, Japan).

*^11^C-ITMM and ^11^C-CB184:* After 15-min irradiation, ^11^C-ITMM [1] and ^11^C-CB184 [2] were prepared using ^11^C-methyl triflate as described previously.

*^18^F-FBPA:* ^18^F-FBPA was prepared using a multipurpose synthesizer CFN-MPS200 (Sumitomo Heavy Industries, Tokyo, Japan) by a slightly modification of a method from a previous report [3]. Briefly, after 120-min irradiation with 25 μA, ^18^F-F_2_ target gas produced above was bubbled into 4 ml trifluoroacetic acid containing 30 mg 4-^10^B-borono-L-phenylalanine at a maximal flow, and then trifluoroacetic acid was removed using a 200 ml/min N_2_ flow heated to 120ºC. The residue was dissolved in 4 ml 0.1% acetic acid and applied to high-performance liquid chromatography: column, YMC-Pack ODS-A (20 mm i.d. ×**︎** 150 mm length, YMC, Kyoto, Japan); mobile phase, 0.1% acetic acid; flow rate, 10 ml/min. ^18^F-FBPA eluted from 16 to 18 min was collected.

**References**

1. Toyohara J, Sakata M, Fujinaga M, Yamasaki T, Oda K, Ishii K, et al. Preclinical and the first clinical studies on [^11^C]ITMM for mapping metabotropic glutamate receptor type 1 by positron emission tomography. Nucl Med Biol 2013;40:214−20

2. Hatano K, Sekimata K, Yamada T, Abe J, Ito K, Ogawa M, et al. Radiosynthesis and i*n vivo* evaluation of two imidazopyridineacetamides, [^11^C]CB184 and [^11^C]CB190, as a PET tracer for 18 kDa translocator protein – direct comparison with [^11^C](R)-PK11195. Ann Nucl Med 2015;29:325–35

3. Ishiwata K, Ido T, Mejia AA, Ichihashi M, Mishima Y. Synthesis and radiation dosimetry of 4-borono-2-[^18^F]fluoro-D,L-phenylalanine: a target compound for PET and boron neutron capture therapy. Appl Radiat Isot 1991;42:325–8.

**Supplementary Table 1** Target folders and target materials

| Production | | Target folder | | | Target foil^1)^ | | Target | |
| --- | --- | --- | --- | --- | --- | --- | --- | --- |
|  | | Material | i.d.^2)^×length | Volume | Material | Thickness | Material^3）^ | Pressure |
|  | |  | mm | ml |  | mm |  | MPa |
| ^11^C-CO_2_ gas | | Aluminum | 32×177 | 137 | Aluminum | 0.6 | N_2_ + O_2_ (0.5%) | 0.8 |
| ^13^N-Ammonium solution | | Niobium | 21×6 | 3.5^4)^ | Haver | 0.050 | H_2_O +10 mM ethanol | 1.8^5)^ |
| ^18^F-Fluoride solution | | Niobium | 21×6 | 3.5^4)^ | Haver | 0.050 | ^18^O-H_2_O^6)^ | 1.8^5)^ |
| ^15^O-Gas | ^15^O-O_2_, ^15^O-CO | Aluminum | 32×183 | 142 | Haver | 0.025 | N_2_ + O_2_ (0.5%) | 0.29 |
|  | ^15^O-CO_2_ |  |  |  |  |  | N_2_ + CO_2_ (2%) |  |
| ^18^F-F_2_ gas | | Aluminum | 32×177 | 137 | Haver | 0.025 | Ne + F_2_ (0.6%) | 0.3 |

^1)^Haver foil (0.01 mm thickness) was used for vacuum side in all target folders. Nominal composition of Haver foil: Co/Cr/Ni/W/Mo/Mn/C/Be/Fe = 42.5%/20.2%/13.0%/2.80%/2.2%/1.60%/0.20%/0.04%/balance. ^2)^Inner diameter. ^3)^Target materials were natural stable isotopes except for ^18^O-H_2_O. ^4)^About 3.6 ml of ^18^O-water was used as the a target and tubing. ^5)^Pressured by He. ^6)^Enriched percentage of ^18^O-H_2_O was ≥98.0 atom%.

**Supplementary Table 2** Radionuclides produced by nuclear reaction using CYPRIS HM-20

|  | Irradiation | | Target | | | Reaction^1)^ | Produced nuclei | | Detected nuclei^2)^ |
| --- | --- | --- | --- | --- | --- | --- | --- | --- | --- |
|  | Beam | MeV^3)^ |  | Isotope^4)^ | Ratio^5)^ |  |  | Half life |  |
| ^11^C-CO_2_ | Proton | 15.8 | N_2_, 99.5% | ^14^N | 9.91E-01 | ^14^N(p,α)^11^C | ^11^C | 20.38 min | ^11^C>^13^N>^14^O |
|  |  |  |  |  |  | ^14^N(p,2α)^7^Be | ^7^Be | 53.24 day |  |
|  |  |  |  |  |  | ^14^N(p,d)^13^N | ^13^N | 9.97 min |  |
|  |  |  |  |  |  | ^14^N(p,n)^14^O | ^14^O | 70.6 sec |  |
|  |  |  |  | ^15^N | 3.62E-03 | ^15^N(p,n)^15^O | ^15^O | 2.04 min |  |
|  |  |  |  |  |  | ^15^N(p,α)^12^C | ^12^C | Stable |  |
|  |  |  | O_2_, 0.5% | ^16^O | 4.99E-03 | ^16^O(p,α)^12^C | ^12^C | Stable |  |
|  |  |  |  |  |  | ^16^O(p,α)^13^N | ^13^N | 9.97 min |  |
|  |  |  |  |  |  | ^16^O(p,d)^15^O | ^15^O | 2.04 min |  |
|  |  |  |  | ^17^O | 1.90E-06 | ^17^O(p,n)^17^F | ^17^F | 64.5 sec |  |
|  |  |  |  |  |  | ^17^O(p,γ)^18^F | ^18^F | 109.8 min |  |
|  |  |  |  | ^18^O | 1.03E-05 | ^18^O(p,n)^18^F | ^18^F | 109.8 min |  |
| ^13^N-Ammonium | Proton | 18.6 | H_2_O  containing  10 mM ethanol | ^16^O | 9.98E-01 | ^16^O(p,pα)^12^C | ^12^C | Stable | ^13^N>^15^O>>^18^F |
|  |  |  |  |  |  | ^16^O(p,α)^13^N | ^13^N | 9.97 min |  |
|  |  |  |  |  |  | ^16^O(p,d)^15^O | ^15^O | 2.04 min |  |
|  |  |  |  | ^17^O | 3.90E-04 | ^17^O(p,n)^17^F | ^17^F | 64.5 sec |  |
|  |  |  |  |  |  | ^17^O(p,γ)^18^F | ^18^F | 109.8 min |  |
|  |  |  |  | ^18^O | 2.04E-03 | ^18^O(p,n)^18^F | ^18^F | 109.8 min |  |
|  |  |  |  | ^12^C | 3.60E-0.4 | ^12^C(p,α)^9^B | ^9^B | 8.0E-18 min |  |
|  |  |  |  |  |  | ^12^C(p,n+p)^11^C | ^11^C | 20.38 min |  |
|  |  |  |  |  |  | ^12^C(p,γ)^13^N | ^13^N | 9.97 min |  |
|  |  |  |  | ^13^C | 3.85E-0.6 | ^13^C(p,n)^13^N | ^13^N | 9.97 min |  |
| ^15^O-CO_2_ | Deuteron | 6.8 | N_2_, 97.5% | ^14^N | 9.71E-01 | ^14^N(d,n)^15^O | ^15^O | 2.04 min | ^15^O>>^13^N |
|  |  |  |  |  |  | ^14^N(d,t)^13^N | ^13^N | 9.97 min |  |
|  |  |  |  | ^15^N | 3.55E-03 | ^15^N(d,p)^16^N | ^16^N | 7.1 sec |  |
|  |  |  | CO_2_, 2.5% | ^12^C | 8.24E-03 | ^12^C(d,n)^13^N | ^13^N | 9.97 min |  |
|  |  |  |  | ^13^C | 8.92E-05 | ^13^C(d,p)^14^C | ^14^C | 5700 year |  |
|  |  |  |  | ^16^O | 1.66E-02 | ^16^O(d,n)^17^F | ^17^F | 64.5 sec |  |
|  |  |  |  | ^18^O | 3.42E-05 | ^18^O(d,α)^16^N | ^16^N | 7.1 sec |  |
|  |  |  |  |  |  | ^18^O(d,p)^19^O | ^19^O | 26.5 sec |  |
| ^15^O-O_2_  ^15^O-CO | Deuteron | 6.8 | N_2_, 99.5% | ^14^N | 9.91E-01 | ^14^N(d,n)^15^O | ^15^O | 2.04 min | ^15^O>>^13^N |
|  |  |  |  |  |  | ^14^N(d,t)^13^N | ^13^N | 9.97 min |  |
|  |  |  |  | ^15^N | 3.62E-03 | ^15^N(d,p)^16^N | ^16^N | 7.1 sec |  |
|  |  |  | O_2_, 0.5% | ^16^O | 4.99E-03 | ^16^O(d,n)^17^F | ^17^F | 64.5 sec |  |
|  |  |  |  | ^18^O | 1.03E-05 | ^18^O(d,α)^16^N | ^16^N | 7.1 sec |  |
|  |  |  |  |  |  | ^18^O(d,p)^19^O | ^19^O | 26.5 sec |  |
| ^18^F-Fluoride | Proton | 18.6 | ^18^O-H_2_O^6)^ | ^18^O | 4.93E-03 | ^18^O(p,n)^18^F | ^18^F | 109.8 min | ^18^F>^17^F>>^13^N |
|  |  |  |  | ^16^O | 6.00E-05 | ^16^O(p,pα)^12^C | ^12^C | Stable |  |
|  |  |  |  |  |  | ^16^O(p,α)^13^N | ^13^N | 9.97 min |  |
|  |  |  |  |  |  | ^16^O(p,d)^15^O | ^15^O | 2.04 min |  |
|  |  |  |  | ^17^O | 1.50E-05 | ^17^O(p,n)^17^F | ^17^F | 64.5 sec |  |
|  |  |  |  |  |  | ^17^O(p,γ)^18^F | ^18^F | 109.8 min |  |
| ^18^F-F_2_ | Deuteron | 7.5 | Ne, 99.4% | ^20^Ne | 9.04E-01 | ^20^Ne(d,α)^18^F | ^18^F | 109.8 min | ^23^Ne>^18^F |
|  |  |  |  |  |  | ^20^Ne(d,n)^21^Na | ^21^Na | 22.50 sec |  |
|  |  |  |  | ^22^Ne | 8.77E-02 | ^20^Ne(d,p)^23^Ne | ^23^Ne | 37.2 sec |  |
|  |  |  | F_2_, 0. 6% | ^19^F | 6.00E-03 | ^19^F(d,n+d)^18^F | ^18^F | 109.8 min |  |

^1)^Possible nuclear reaction based on OECD NEA Data Bank (http://www.oecd-nea.org/janis/book/). ^2)^Detected radionuclides in this study by measuring radioactivity decay in the order of amounts at the end of irradiation. ^3)^Incident energy. ^4)^Ratios of natural isotopes: ^12^C vs ^13^C, 98.93% vs 1.07%; ^14^N vs ^15^N, 99.64% vs 0.36%; ^16^O vs ^17^O vs ^18^O, 99.757% vs 0.038% vs 0.205%; ^20^Ne vs ^21^Ne vs ^22^Ne, 90.92% vs 0.26 vs 8.82%, respectively. ^5)^Ratio of each isotopes included in the target. ^6)^Ratios of isotopes: ^18^O vs ^17^O vs ^16^O, 98.5% vs 0.3% vs 1.2%.

**Supplementary Table 3** Percentages of positron-emitting nuclides in ^11^C-CO_2_ target gas and ^11^C-labeled compounds detected by measuring radioactivity decay

|  | Irradiation | | ^11^C | ^13^N | ^14^O |
| --- | --- | --- | --- | --- | --- |
|  | μAh | n | % | | |
| ^11^C-CO_2_ target gas^1)^ | 2.5 | 3 | 47.3  (43.6–50.3) | 28.5  (24.2–32.9) | 24.2  (16.8–32.2) |
| ^11^C-CO_2_ gas^2)^ | 2.5 | 3 | 100.0 |  |  |
| ^11^C-CH_3_I^2)^ | 2.5 | 3 | 100.0 |  |  |
| ^11^C-Methionine^3)^ | 6.25 | 1 | 100.0 |  |  |
| ^11^C-ITMM^3)^ | 12.5 | 2 | 100.0 |  |  |
| ^11^C-CB184^3)^ | 12.5 | 1 | 100.0 |  |  |

Irradiation was expressed as integrated current. Immediately after 30-sec recovery of the ^11^C-CO_2_ target gas, recovery of ^11^C-CO_2_ gas and ^11^C-CH_3_I or synthesis of ^11^C-labeled tracers, radioactivity was measured for 5–8 h until reaching to background levels using a radioisotope calibrator. Percentages (average with range in parenthesis) of radionuclides were decay-corrected at the end of irradiation^1)^, at the time of recovery^2)^ or the end of synthesis^2)^.

**Supplementary Table 4** Percentages of positron-emitting chemicals in ^11^C-CO_2_ target gas detected by gas chromatography

|  | Irradiation | | ^11^C-CO_2_ | ^11^C-CO | ^14^O-O_2_ | ^13^N-N_2_ | ^13^N-N_2_O |
| --- | --- | --- | --- | --- | --- | --- | --- |
|  | μAh | n | % | | | | |
| ^11^C-CO_2_ target gas | 5.0  (1.25–12.5) | 4 | 60.4  (52.4–65.3) | 1.0  (0.8–1.2) | 4.9  (2.3–6.2) | 33.6  (29.9–40.2) | 0.1  (0.0–0.2) |

Irradiation was expressed as integrated current (average with range in parenthesis). To confirm minor components, integrated current was increased until 12.5 μAh. Percentages of three components, ^14^O-O_2_, ^13^N-N_2_, and ^11^C-CO, on the Molecular Sieves 13X column were decay-corrected at the start of analysis, and considering these ratios and percentages of each components on the Porapak Q column, the percentages (average with range in parenthesis) of ^11^C-CO_2_, ^11^C-CO, ^14^O-O_2_, ^13^N-N_2_, and ^13^N-N_2_O were calculated and decay-corrected at the start of analysis.

**Supplementary Table 5** Percentages of radionuclides in ^15^O-labeled compounds detected by measuring radioactivity decay

|  | Irradiation | | ^15^O | ^13^N |
| --- | --- | --- | --- | --- |
|  | μAh | n | % | |
| ^15^O-CO_2_ | 1.7 | 4 | 99.3 | 0.7 |
| ^15^O-O_2_ | 1.9  (1.7–2.5) | 4 | 99.4  (99.4–99.5) | 0.6  (0.5–0.6) |
| ^15^O-CO | 1.7 | 4 | 99.4  (99.3–99.4) | 0.6  (0.6–0.7) |
| ^15^O-H_2_O | 6.1  (1.7–8.3) | 3 | 100.0 |  |

Irradiation was expressed as integrated current (average with range in parenthesis). Immediately after 30-sec recovery ^15^O-labeled gases and ^15^O-H_2_O, radioactivity was measured for about 130 min until reaching to background levels using a radioisotope calibrator. Percentages (average with range in parenthesis) of radionuclides were decay-corrected at the end of recovery.

**Supplementary Table 6** Percentages of positron-emitting chemicals in ^15^O-gases detected by gas chromatography

|  | Irradiation | | ^15^O-O_2_ | ^15^O-CO | ^15^O-CO_2_ | ^13^N-N_2_ |
| --- | --- | --- | --- | --- | --- | --- |
|  | μAh | n | % | | | |
| ^15^O-CO_2_ | 3.1  (1.7–5.0) | 6 |  | 0.5  (0.3–0.9) | 98.8  (98.3–99.1) | 0.7  (0.5–0.8) |
| ^15^O-O_2_ | 1.4  (0.8–1.7) | 3 | 99.4  (99.3–99.5) |  |  | 0.6  (0.5–0.7) |
| ^15^O-CO | 1.5  (0.8–1.7) | 6 |  | 99.3  (99.2–99.4) |  | 0.7  (0.6–0.8) |

Irradiation was expressed as integrated current (average with range in parenthesis). To confirm minor components, integrated current was increased until 5.0 μAh. Percentages of three components, ^15^O-O_2_, ^13^N-N_2_, and ^15^C-CO, on the Molecular Sieves 13X column were decay-corrected at the start of analysis, and considering these ratios and percentages of each components on the Porapak Q column, the percentages (average with range in parenthesis) of ^15^O-O_2_, ^15^C-CO, ^15^C-CO_2_, and ^13^N-N_2_ were calculated and decay-corrected at the start of analysis.

**Supplementary Table 7** Percentages of positron-emitting nuclides in ^18^F-F_2_ target gas and ^18^F-FBPA detected by measuring radioactivity decay

|  | Irradiation | | ^18^F | ^23^Ne |
| --- | --- | --- | --- | --- |
|  | μAh | n | % | |
| ^18^F-F_2_ target gas ^1)^ | 9.2  (2.5–12.5) | 3 | 16.8  (16.6–17.1) | 83.2  (82.9–83.4) |
| ^18^F-FBPA^2)^ | 50 | 1 | 100.0 |  |

Irradiation was expressed as integrated current (average with range in parenthesis). Immediately after 60-sec recovery of ^18^F-F_2_ target gas or after the end of synthesis, radioactivity was measured for 5–22 h until it was less than 0.5 MBq using a radioisotope calibrator. Percentages (average with range in parenthesis) of radionuclides were decay-corrected at the end of irradiation^1)^ or the end of synthesis^2)^.

**Supplementary Table 8** Percentages of positron-emitting nuclides in ^13^N-ammonium target solution and ^13^N-NH_3_ detected by measuring radioactivity decay

|  | Irradiation | | ^13^N | ^15^O | ^18^F |
| --- | --- | --- | --- | --- | --- |
|  | μAh | n | % | | |
| ^13^N-Ammonium target solution^1)^ | 10.6  (1.67–25) | 3 | 58.3  (54.2–61.4) | 41.1  (37.8–45.2) | 0.6  (0.3–0.9) |
| ^13^N-NH_3_^2)^ | 4.44  (1.67–8.33) | 3 | 100.0 |  | 0.0^3)^ |

Irradiation was expressed as integrated current (average with range in parenthesis). To confirm minor components, integrated current was increased until 25 μAh. Immediately after recovery of the ^13^N-ammonium target solution or after the synthesis of ^13^N-NH_3_, radioactivity was measured for 5–19 h until it was less than 0.6 MBq using a radioisotope calibrator. Percentages (average with range in parenthesis) of radionuclides were decay-corrected at the end of irradiation^1)^ or the end of synthesis^2)^. ^3)^Very small amounts of ^18^F were detected: 0.005% (range, 0.004–0.008%) at the end of synthesis.

**Supplementary Table 9** Percentages of positron-emitting chemicals in ^13^N-ammonium target solution and ^13^N-NH_3_ detected by ion chromatography

|  | Irradiation | | Chemicals | | | | |
| --- | --- | --- | --- | --- | --- | --- | --- |
|  | μAh | n | % | | | | |
| Anion-exchange column:  Shim-pack IC-SA2 | | | Void-1 | Fluoride | Unknown-1 | Nitrite | Nitrate |
|  |  |  | 2.2 min | 3.9 min | 4.5 min | 6.3 min | 8.9 min |
|  |  |  | ^13^N | ^18^F | ^13^N | ^13^N | ^13^N |
| ^13^N-Ammonium target solution^3)^ | 17.5  (12.5–25) | 5 | 96.5^1)^  (94.9–98.7) | 1.9  (1.1–2.7) | 0.8 ^1)^  (0.0–2.2) | 0.6^1)^  (0.0–1.0) | 0.2^1)^  (0.0–0.8) |
| ^13^N-NH_3_^4)^ | 1.67 | 3 | 100.0 | ND^5)^ |  |  |  |
| Cation-exchange column:  Shim-pack IC-C4 | | | Void-2^2)^ | | Unknown-2^2)^ | | Ammonium |
|  |  |  | 1.8 min | | 2.0 min | | 5.8 min |
|  |  |  | (^13^N) | (^18^F) | (^13^N) | (^18^F) | ^13^N |
| ^13^N-Ammonium target solution^3)^ | 12.5 | 3 | 0.2  (0.1–0.4) | 0.1  (0.0–0.1) | 6.3  (3.6–9.4) | 1.3  (0.2–1.9) | 92.1  (88.2–94.4) |
| ^13^N-NH_3_^4)^ | 3.61  (3.33–4.17) | 3 |  |  |  |  | 100.0 |

Irradiation was expressed as integrated current (average with range in parenthesis). To confirm minor components, integrated current was increased until 25 μAh. The ^13^N-ammonium target solutions and ^13^N-NH_3_ were successively analyzed once to five times at 4–6 min to 58–73 min after the end of irradiation and at 2–8 min to 60–65 min after the end of synthesis, respectively. ^1)^Because all minor peaks were disappeared by the second analysis (14–25 min), they were assumed to be labeled with ^13^N. ^2)^Based on the hypothesis that ^13^N-components in two minor peaks disappeared by the fifth analysis (58–67 min), the ratios of ^13^N- and ^18^F-components were separately evaluated. Percentages (average with range in parenthesis) of each components analyzed at the first time were decay-corrected at the end of irradiation^3)^ or the end of synthesis^4)^. Because ^15^O-radioactivity detected by measuring radioactivity decay could not be assigned in any radioactive peaks, ^15^O-radioactivity was ignored in this analysis. ^5)^ND: Although a very small amount of ^18^F-component (0.005%) was detected by measuring radioactivity decay (Supplementary Table 8), no minor component was detected by ion chromatography, suggesting that the ^18^F-component was below detection limit.

**Supplementary Table 10** Percentages of positron-emitting nuclides in ^18^F-fluoride target solution, ^18^F-NaF, and ^18^F-FDG detected by measuring radioactivity decay

|  | Irradiation | | ^18^F | ^13^N | ^17^F |
| --- | --- | --- | --- | --- | --- |
|  | μAh | n | % | | |
| ^18^F-Fluoride target solution^1)^ | 37.5 | 3 | 87.2  (86.6–89.6) | 0.8  (0.6–0.9) | 12.0  (9.5–13.7) |
| ^18^F-NaF^2)^ | 7.5 | 4 | 99.5  (99.3–99.6) | 0.5  (0.4–0.7) |  |
| ^18^F-FDG^2)^ | 27.5  (7.5–37.5) | 3 | 100.0 |  |  |

Irradiation was expressed as integrated current (average with range in parenthesis). Immediately after recovery of the ^18^F-fluoride target solution or after the synthesis of ^18^F-NaF and ^18^F-FDG, radioactivity was measured for 22–29 h until it was less than 10 MBq using a radioisotope calibrator. Percentages (average with range in parenthesis) of radionuclides were decay-corrected at the end of irradiation^1)^ or the end of synthesis^2)^.

**Supplementary Table 11** Percentages of positron-emitting chemicals in ^18^F-fluoride target solution, ^18^F-NaF, and ^18^F-FDG detected by ion chromatography

|  | Irradiation | | Chemicals | | | | | |
| --- | --- | --- | --- | --- | --- | --- | --- | --- |
|  | μAh | n | % | | | | | |
| Anion-exchange column:  Shim-pack IC-SA2 | | | Void-1 | FDG | Unknown-1 | Fluoride | Nitrite | Nitrate |
|  |  |  | 2.2 min | 2.5 min | 3.2 min | 3.9 min | 6.3 min | 8.9 min |
|  |  |  |  | ^18^F | ^18^F | ^18^F | ^13^N | ^13^N |
| ^18^F-Fluoride target  solution^2)^ | 37.5 | 3 |  |  |  | 99.4  (99.0–100.0) |  | 0.6  (0.0–1.0) |
| ^18^F-NaF^3)^ | 6.1  (1.0–11.3) | 3 |  |  |  | 98.3  (96.3–99.9) | 0.2  (0.1–0.4) | 1.5  (0.1–3.5) |
| ^18^F-FDG^3)^ | 12.8  (5.8–16.7) | 5 |  | 96.9  (96.3–97.3) | 0.9  (0.8–1.1) | 2.2  (1.9–2.6) |  |  |
| Cation-exchange column:  Shim-pack IC-C4 | | | Void-2^1)^ | | Fluoride/FDG | Unknown-2 |  |  |
|  |  |  | 1.8 min | | 2.0 min | 2.3 min |  |  |
|  |  |  | (^13^N) | (^18^F) | ^18^F | ^18^F |  |  |
| ^18^F-Fluoride target  solution^2)^ | 12.5  (1.7–23.3) | 2 | 1.3  (0.0–2.6) | 2.1  (0.7–3.6) | 96.6  (96.4–96.7) |  |  |  |
| ^18^F-NaF^3)^ | 5.7  (1.7–9.8) | 2 |  |  | 100.0 |  |  |  |
| ^18^F-FDG^3)^ | 20.2  (10–37.5) | 4 |  |  | 97.6  (96.3–99.5) | 2.4  (0.5–3.7) |  |  |

Irradiation was expressed as integrated current (average with range in parenthesis). To confirm minor components, integrated current was decreased until 1.0 μAh. ^18^F-fluoride target solutions were successively analyzed three times at 5–8 min to 71 min after the end of irradiation. Considering the 5–8 min and retention time of fluoride, ^17^F-radioactivity (t_1/2_ = 64.5 sec) in the ^18^F-fluoride target solutions was negligible. Therefore, contribution of ^17^F could not be estimated in ion chromatography analysis. ^1)^Based on the hypothesis that ^13^N-components in the minor peaks disappeared by the fifth analysis (58–67 min), the ratios of ^13^N- and ^18^F-components were evaluated separately. Percentages (average with range in parenthesis) of each component analyzed at the first time were corrected at the end of irradiation^2)^ or the end of synthesis^3)^. Because ^17^F-radioactivity detected by measuring radioactivity decay could not be assigned in any radioactive peaks, the ^17^F-radioactivity was ignored in this analysis.

**Supplementary Table 12** Presence of longer half-life γ-ray emitting nuclides in target solutions and positron-emitting compounds detected using a high-purity Ge detector

|  | Irradiation | | Product | Ratios against ^18^F, ^13^N, or ^11^C | | | | | | |
| --- | --- | --- | --- | --- | --- | --- | --- | --- | --- | --- |
|  |  |  |  | Cr-51 | Mn-52 | Mn-54 | Co-55 | Co-56 | Co-57 | Co-58 |
|  | μAh | n | GBq | (27.7 d) | (5.59 d) | (312 d) | (15.7 h) | (77.2 d) | (272 d) | (70.9 d) |
| ^13^N-Ammonium target solution^1)^ | 25 | 3 | 13.5 | 3.39E-7 | 2.13E-7 | 3.14E-9 | 2.31E-6 | 3.27E-8 | 3.36E-8 | 4.05E-7 |
| ^13^N-NH_3_^2)^ | 3.3 | 3 | 7.4 | 1.25E-12 | 1.95E-13 | ND | 1.14E-14 | 6.33E-14 | ND | 4.06E-13 |
| ^18^F-Fluoride target solution^1)^ | 37.5 | 3 | 93.8 | 6.73E-8 | 2.14E-8 | 4.06E-10 | 1.38E-8 | 3.14E-10 | 4.12E-10 | 4.49E-9 |
| ^18^F-NaF^2)^ | 37.5 | 3 | 78.3 | 7.84E-8 | ND | 1.70E-11 | ND | 2.29E-11 | ND | ND |
| ^18^F-FDG^2)^ | 37.5 | 3 | 63.4 | ND | ND | ND | ND | 2.09E-10 | ND | ND |
| ^11^C-Methionine^2)^ | 2.5 | 1 | 2.9 | ND | ND | ND | ND | ND | ND | ND |
| ^18^F-FBPA^2)^ | 50 | 3 | 0.53 | ND | ND | ND | ND | ND | ND | ND |

Irradiation was expressed as integrated current (average with range in parenthesis). The irradiation for ^13^N-NH_3_ was suitable for clinical use; however, the integrated current for ^13^N-ammonium target solution was much increased for comparison with ^18^F-fluoride target solution. Radionuclides with a half-life in parenthesis were estimated as ratios against total radioactivity of ^18^F, ^13^N or ^11^C at the end of irradiation^1)^ or the end of synthesis^2)^.

**Supplementary Table 12** (Continued)

|  | Irradiation | | Product | Ratios against ^18^F, ^13^N, and ^11^C | | | | | |
| --- | --- | --- | --- | --- | --- | --- | --- | --- | --- |
|  |  |  |  | Ni-57 | Ga-67 | Mo-93m | Tc-95 | Tc-96 | Re-181 |
|  | μAh | n | GBq | (1.48 d) | (3.26 d) | (6.85 h) | (20.0 h) | (4.28 d) | (19.9 h) |
| ^13^N-Ammonium target solution^1)^ | 25 | 3 | 13.5 | 9.11E-7 | ND | ND | 6.76E-8 | 9.94E-9 | 4.13E-8 |
| ^13^N-NH_3_^2)^ | 3.3 | 3 | 7.4 | NE | ND | NE | NE | ND | NE |
| ^18^F-Fluoride target solution^1)^ | 37.5 | 3 | 93.8 | 3.30E-9 | 1.55E-9 | 7.03E-8 | 1.89E-8 | 2.93E-9 | 3.75E-9 |
| ^18^F-NaF^2)^ | 37.5 | 3 | 78.3 | ND | ND | 6.29E-8 | 1.14E-8 | 1.85E-9 | 3.59E-9 |
| ^18^F-FDG^2)^ | 37.5 | 3 | 63.4 | ND | ND | ND | 1.45E-9 | 2.53E-10 | 7.94E-9 |
| ^11^C-Methionine^2)^ | 2.5 | 1 | 2.9 | ND | ND | ND | ND | ND | ND |
| ^18^F-FBPA^2)^ | 50 | 3 | 0.53 | ND | ND | ND | ND | ND | ND |

Irradiation was expressed as integrated current (average with range in parenthesis). The irradiation for ^13^N-NH_3_ was suitable for clinical use; however, the integrated current for ^13^N-ammonium target solution was much increased for comparison with ^18^F-fluoride target solution. Radionuclides with a half-life in parenthesis were estimated as ratios against total radioactivity of ^18^F, ^13^N or ^11^C at the end of irradiation^1)^ or the end of synthesis^2)^.


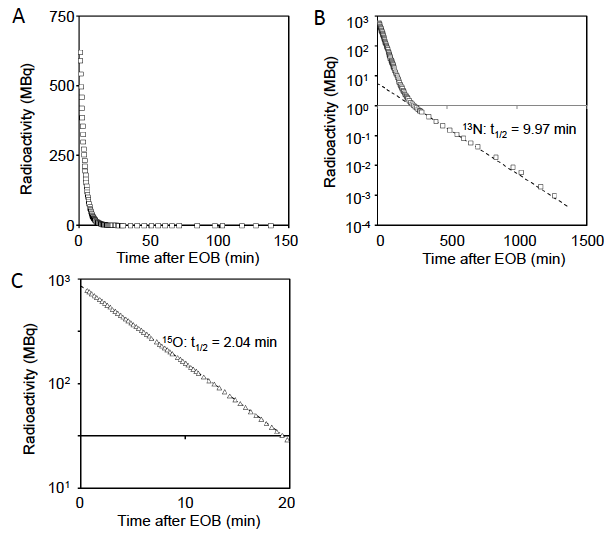


**Supplementary Fig. 1** Radioactivity decay curve of ^15^O-CO_2_.

A) Total radioactivity decay (square) on a linear scale. B) Total radioactivity decay (square) on a log scale. The later phase indicates a half-life of ^13^N. The ^13^N-radioactivity was extrapolated to time zero and subtracted from total radioactivity. C) The residual radioactivity decay (triangle) on a log scale indicates a half-life of ^15^O.


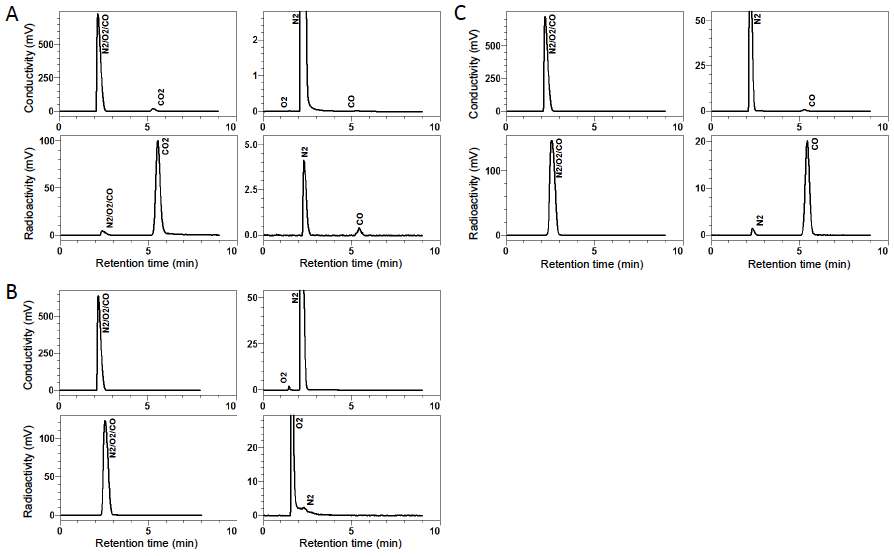


**Supplementary Fig. 2** Gas chromatograms of ^15^O-CO_2_ (A), ^15^O-O_2_ (B), and ^15^O-CO (C).

Left side: analysis on a Porapak Q column; right side: analysis on a Molecular Sieve 13X column. Upper row: thermal conductivity; lower row, radioactivity.


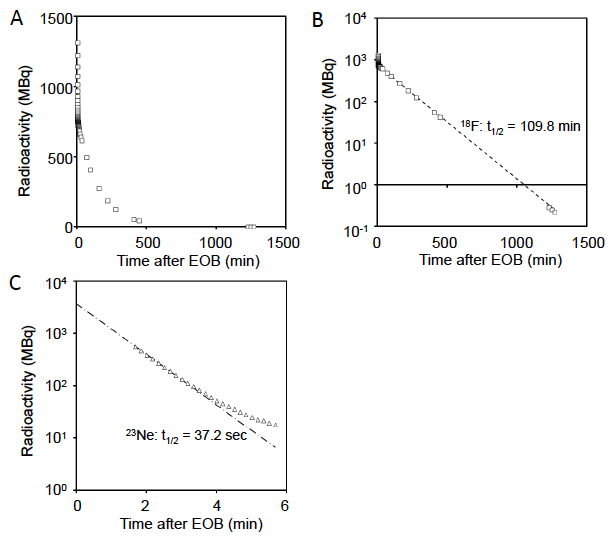


**Supplementary Fig. 3** Radioactivity decay curve of ^18^F-F_2_ target gas.

A) Total radioactivity decay (square) on a linear scale. B) Total radioactivity decay (square) on a log scale indicates a half-life of ^18^F. The ^18^F-radioactivity was extrapolated to time zero and subtracted from total radioactivity. C) The residual radioactivity decay (triangle) on a log scale indicates a half-life of ^23^Ne.


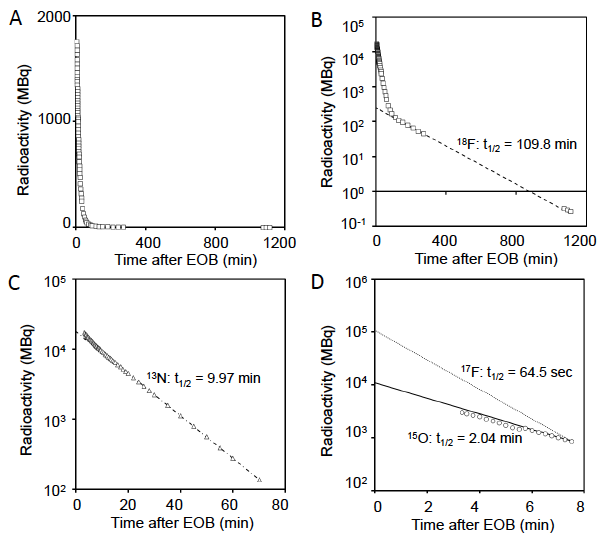


**Supplementary Fig. 4** Radioactivity decay curves of ^13^N-ammonium target solution.

A) Total radioactivity decay (square) on a linear scale. B) Total radioactivity decay (square) on a log scale. The later phase indicates a half-life of ^18^F. The ^18^F-radioactivity was extrapolated to time zero and subtracted from total radioactivity. C) The residual radioactivity decay (triangle) on a log scale indicates a half-life of ^13^N. The ^13^N-radioactivity was further subtracted from the residual radioactivity. D) The final radioactivity decay (circle) on a log scale indicates a half-life of ^15^O.


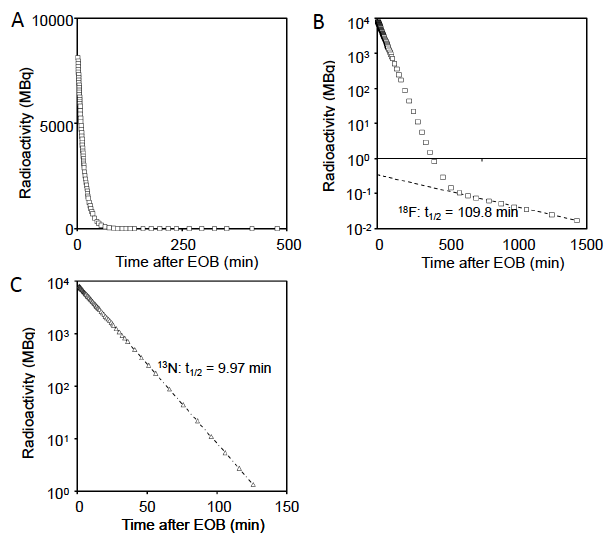


**Supplementary Fig. 5** Radioactivity decay curves of ^13^N-NH_3_.

A) Total radioactivity decay (square) on a linear scale. B) Total radioactivity decay (square) on a log scale. The later phase indicates a half-life of ^18^F. The ^18^F-radioactivity was extrapolated to time zero and subtracted from total radioactivity. C) The residual radioactivity decay (triangle) on a log scale indicates a half-life of ^13^N.


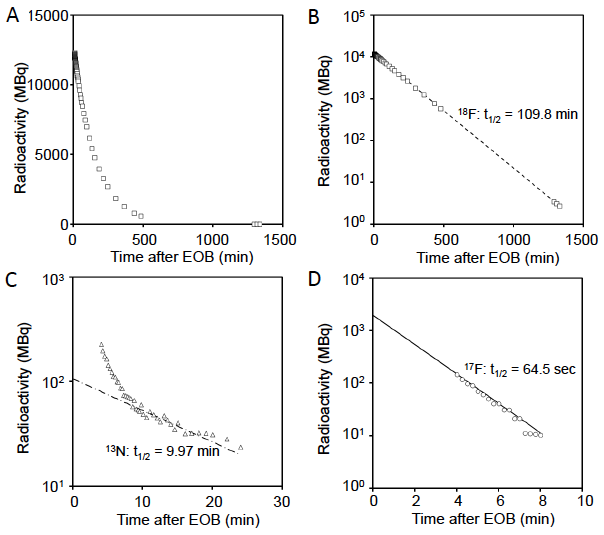


**Supplementary Fig. 6** Radioactivity decay curves of ^18^F-fluoride target solution.

A) Total radioactivity decay (square) on a linear scale. B) Total radioactivity decay (square) on a log scale indicates a half-life of ^18^F. The ^18^F-radioactivity was extrapolated to time zero and subtracted from total radioactivity. C) The later phase of residual radioactivity decay (triangle) on a log scale indicates a half-life of ^13^N. The ^13^N-radioactivity was further subtracted from the residual radioactivity. D) The final radioactivity decay (circle) on a log scale indicates a half-life of ^17^F.


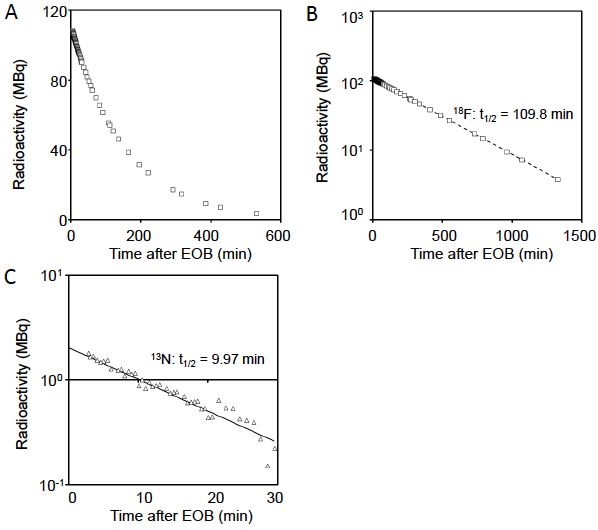


**Supplementary Fig. 7** Radioactivity decay curves of ^18^F-NaF.

A) Total radioactivity decay (square) on a linear scale. B) Total radioactivity decay (square) on a log scale indicates a half-life of ^18^F. The ^18^F-radioactivity was extrapolated to time zero and subtracted from total radioactivity. C) The residual radioactivity decay (triangle) on a log scale indicates a half-life of ^13^N.


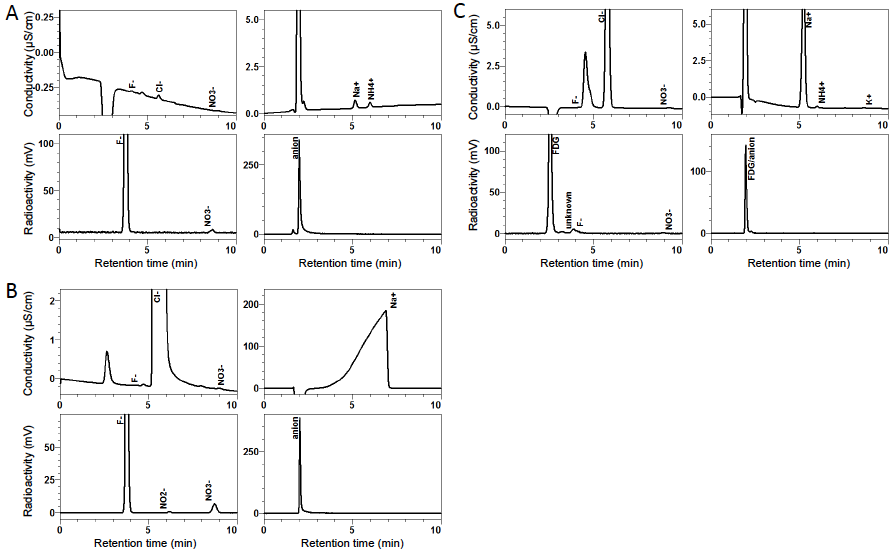


**Supplementary Fig. 8** Ion chromatograms of ^18^F-fluoride target solution (A), ^18^F-NaF (B), and ^18^F-FDG (C).

Left side: analysis on an anion-exchange Shim-pack IC-SA2 column; right side: analysis on a cation-exchange Shim-pack IC-C4 column. Upper row of each: conductivity; lower row of each, radioactivity.
